# The Emerging Uncommon Non-Albicans Candida: Candida Parapsilosis Peritonitis in a Peritoneal Dialysis Patient

**DOI:** 10.7759/cureus.17083

**Published:** 2021-08-11

**Authors:** Sasmit Roy, Praveena Vantipalli, Amarinder Garcha, Mytri Pokal, Sreedhar Adapa

**Affiliations:** 1 Nephrology, University of Virginia, Lynchburg, USA; 2 Nephrology, Liberty University Medical School, Lynchburg, USA; 3 Family Medicine, University of Nebraska Medical Center, Omaha, USA; 4 Nephrology, Kaweah Delta Medical Center, Visalia, USA; 5 Internal Medicine, Mercer University School of Medicine, Macon, USA

**Keywords:** candida parapsilosis, peritonitis, peritoneal dialysis, invasive fungal infection, end stage renal disease

## Abstract

*Candida parapsilosis* can cause invasive fungal infection which is associated with significant morbidity and mortality. Timely management of this uncommon Candida pathogen is essential to prevent peritoneal dialysis patients from succumbing to the infectious complications of peritonitis related to it. We present a 75-year-old Caucasian female with end-stage renal disease, on peritoneal dialysis at home, who presented with peritonitis features found to be related to this rare Candida species. She was treated with four weeks course of oral fluconazole and was switched to incenter hemodialysis. Physicians need to be aware of this notorious Candida species in peritoneal dialysis patients and prompt management is essential in successful patient outcomes.

## Introduction

*Candida parapsilosis* (*C. parapsilosis*) causes invasive fungal infections and is widely distributed in nature. Invasive fungal infection carries high morbidity and mortality risk to all infected patients. Fungal peritonitis itself is associated with serious complications in patients receiving peritoneal dialysis. Invasive fungal peritonitis mandates removal of peritoneal dialysis catheter and switches to hemodialysis modality till the fungal infection has completely resolved. We present a case of an end-stage renal disease patient on peritoneal dialysis, who developed peritonitis caused by *C. parapsilosis* and was managed with antifungals followed by catheter removal.

## Case presentation

A 75-year-old elderly Caucasian female with end-stage renal disease (ESRD), who was on continuous ambulatory peritoneal dialysis (CAPD) for the past three years, was admitted to the hospital with the chief complaint of worsening diffuse abdominal pain, nausea, and low-grade fever of three days duration. The abdominal pain was diffuse; eight of 10 in intensity, dull aching in type. She was having pain with each peritoneal dialysis exchange at home. The patient denies any vomiting, diarrhea, constipation, chills, or rigors. Past medical history was significant for type 2 diabetes mellitus, essential hypertension, and hyperlipidemia. She had no history of prior peritonitis. Her home medications included glipizide 5 milligrams (mg) daily, lisinopril 40 mg daily, carvedilol 25 mg twice a day, aspirin 81 mg daily, atorvastatin 40 mg daily, calcitriol 0.5 μg daily, and daily renal vitamin. Her CAPD prescription was four exchanges during daytime with 2.5 % dextrose solution, with 2 L in each dialysate and for 4 hours each exchange. She was essentially anuric.

The vital signs on presentation were as follows: temperature of 99.5 degrees Fahrenheit (°F), pulse rate of 77 beats per minute, blood pressure of 130/70 mm Hg, and respiratory rate of 22 breaths per minute with oxygen saturation of 99% on room air. The physical examination revealed that the patient was in moderate painful distress. Abdominal examination revealed diffuse tenderness but no rigidity. There were no peritoneal signs or fluid thrill present. Bowel sounds were audible in all four quadrants. The Tenckhoff peritoneal dialysis catheter exit site in the left lower quadrant was clean with no tenderness or discharge visible. The rest of the systemic physical examination was insignificant.

The laboratory data showed white blood cell (WBC) count 9330/mm^3^, hemoglobin 9.1 g/dL, platelets 119,000/ mm^3^, sodium 127 mmol/L, potassium 3.9 mmol/L, blood urea nitrogen 44 mg/dL, and serum creatinine 6.61 mg/dL (Table 1). The peritoneal dialysis (PD) fluid analysis revealed a WBC count of 595 cells/μL, with 76% predominant neutrophils. Peritoneal fluid Gram stain revealed >100 WBC, and no organisms were seen. Chest x-ray was unremarkable. Computed axial tomography of the abdomen didn’t reveal any other source of intra-abdominal pathology. The patient was started on empiric intra-peritoneal broad-spectrum antibiotics, namely vancomycin and ceftazidime in an outpatient setting for suspected peritonitis two days prior, because of moderate abdominal pain. She was also started on p.o fluconazole for fungal prophylaxis. The peritoneal dialysis (PD) fluid culture grew *C. parapsilosis* in the aerobic bottle after three days of incubation. Intraperitoneal vancomycin and ceftazidime were stopped after the culture results and the patient was continued on treatment with fluconazole 200 mg daily for a total of four weeks duration. The PD catheter was removed for unremitting abdominal pain and as per guidelines for removal of the catheter in cases with fungal peritonitis. The dialysis modality was changed to thrice weekly in-center hemodialysis. She was discharged by day five of admission with outpatient dialysis unit follow-up. By four weeks of antifungal treatment, her symptoms resolved with no further abdominal pain or discomfort. She was resumed on peritoneal dialysis after four months and thereafter she has been doing uninterrupted peritoneal dialysis at home with no recurrence of fungal infection.

**Table 1:** Laboratory data on admission ALP: alkaline phosphatase; AST: serum aspartate aminotransferase; ALT: serum alanine aminotransferase

	Reference Range	On Presentation
Sodium (mmol/L)	135-145	127
Potassium (mmol/L)	3.5-5.1	4.2
Chloride (mmol/L)	98-106	96
CO_2_ (mmol/L)	23-29	23
Anion Gap (mmol/L)	8-14	10
Blood Urea Nitrogen (BUN) (mg/dL)	8-24	44
Creatinine (Cr) (mg/dL)	0.7-1.3	6.61
Calcium (mg/dL)	8.8-10.2	8.3
Glucose (mg/dL)	70-105	102
ALP (IntUnit/L)	45-115	56
AST (IntUnit/L)	8-48	22
ALT (IntUnit/L)	7-55	24
White Blood Cells (k/mm^3^)	4-10	9.1
Hemoglobin (g/dL)	14-16	9.1
Hematocrit (%)	42-51	29
Platelets (k/mm^3^)	150-450	119
Peritoneal Fluid Cell Count (cells/μL)	<300	595

## Discussion

Infectious peritonitis is still a major complication challenging patients, who undergo peritoneal dialysis. In this age of US federal government impetus towards home dialysis, peritonitis remains as a major impediment and a leading cause of peritoneal dialysis failure forcing the patients to switch to incenter hemodialysis [1]. Although less than 5% of peritonitis episodes result in death, in around 16% of PD patients, peritonitis is the direct or major reason for death. In addition, prolonged or severe peritonitis contributes to functional and structural alterations of the peritoneal membrane, ultimately leading to membrane failure [1].

Although the vast majority of cases are due to a bacterial origin, approximately 3% to 6% are due to fungal infections, more specifically Candida species [2]. Although *Candida albicans* is the commonest Candida species identified, *C. parapsilosis* is fast emerging in a significant majority of these cases [3]. In a retrospective study over nine years among 890 CAPD patients in Hong Kong, there were 70% of fungal peritonitis of which 50% were caused specifically by *C. parapsilosis* [3]. Multiple studies worldwide including those done in Taiwan, Mexico, the United Kingdom, and Israel have found *C. parapsilosis* to be a very common and deadly infectious etiology of fungal peritonitis among the patients on peritoneal dialysis [4-7].

*C. parapsilosis* was first isolated from a diarrhea patient in Puerto Rico in 1928 [8] and is now considered the second leading cause of invasive candidiasis in the United States [9]. It has a much higher complication rate compared to *C. albicans *and thereby mandates more aggressive treatment and preventive measures [10]. It is a common skin flora and a subungual proliferator. Improper performance of sterile technique when handling the catheter tip and dialysate bag, cutaneous site of catheter entry, or transmigration across the bowel wall can all be points of entry for infection. Almost all cases of fungal peritonitis have a recent history of bacterial peritonitis or recent antibacterial use [11-14]. Other reported and potential risk factors being abdominal surgery, extraperitoneal fungal infection, use of emergency (urgent start) peritoneal dialysis, human immunodeficiency virus (HIV) infection, etc. [11-14].

When *C. parapsilosis* is introduced to a high glucose solution, it can produce excessive slime material which can act as a biofilm [15]. Given that dialysate fluid is high in glucose concentration and the pathogen can produce a biofilm that enhances adherence to a plastic peritoneal dialysis catheter, could be the reason *C. parapsilosis* is so prevalent in PD fungal infections. Fungal peritonitis itself carries higher morbidity and mortality, complications can include abscess formation, sclerosing peritonitis, adhesions causing bowel wall strictures or obstructions or perforation, etc. [16].

The commonest symptoms and signs of fungal peritonitis are similar to bacterial peritonitis in form of abdominal discomfort and cloudy dialysate. Abdominal pain may be diffuse with diarrhea/vomiting/nausea, or it can be discrete with only mild pyrexia. Cell count of peritoneal fluid with > 100 WBC with polymorphonuclear cell predominance along with Gram stain predominant with yeasts and culture of fluid are the standard method of diagnosis.

Multiple observational studies have recommended prophylaxis with either nystatin (400,000 to 500,000 units thrice daily) or fluconazole (200 mg every other day or 100 mg daily) for the duration of antibiotics for bacterial peritonitis to prevent this dreadful complication [17,18]. Based on these findings, our patient was started on oral fluconazole as antifungal prophylaxis. According to the International Society of Peritoneal Dialysis for fungal peritonitis in 2016, these fungal peritonitis patients should be treated with fluconazole along with immediate catheter removal and transition to hemodialysis [1]. Oral fluconazole is recommended to treat Candida species due to its high bioavailability compared to IV fluconazole. Treatment duration is usually two to four weeks. 

## Conclusions

Our case highlights that in peritoneal dialysis patients clinicians should be aware of the importance of still rare yet dreadful species Candida. To avoid the serious morbidity and mortality associated with it, prompt early diagnosis and treatment are required. Patients can return to peritoneal dialysis mode once the fungal infection clears and clinical signs or symptoms of peritonitis are absent.
